# Striatal Synapse Degeneration and Dysfunction Are Reversed by Reactivation of Wnt Signaling

**DOI:** 10.3389/fnsyn.2021.670467

**Published:** 2021-06-03

**Authors:** Soledad Galli, Stefka H. Stancheva, Tom Dufor, Alasdair J. Gibb, Patricia C. Salinas

**Affiliations:** ^1^Department of Cell and Developmental Biology, University College London, London, United Kingdom; ^2^Department of Neuroscience, Physiology and Pharmacology, University College London, London, United Kingdom

**Keywords:** synapse degeneration, Dkk1, neuronal circuit restoration, medium spiny neurons, neurodegenerative disease

## Abstract

Synapse degeneration in the striatum has been associated with the early stages of Parkinson’s and Huntington’s diseases (PD and HD). However, the molecular mechanisms that trigger synaptic dysfunction and loss are not fully understood. Increasing evidence suggests that deficiency in Wnt signaling triggers synapse degeneration in the adult brain and that this pathway is affected in neurodegenerative diseases. Here, we demonstrate that endogenous Wnt signaling is essential for the integrity of a subset of inhibitory synapses on striatal medium spiny neurons (MSNs). We found that inducible expression of the specific Wnt antagonist Dickkopf-1 (Dkk1) in the adult striatum leads to the loss of inhibitory synapses on MSNs and affects the synaptic transmission of D2-MSNs. We also discovered that re-activation of the Wnt pathway by turning off Dkk1 expression after substantial loss of synapses resulted in the complete recovery of GABAergic and dopamine synapse number. Our results also show that re-activation of the Wnt pathway leads to a recovery of amphetamine response and motor function. Our studies identify the Wnt signaling pathway as a potential therapeutic target for restoring neuronal circuits after synapse degeneration.

## Introduction

Synapse degeneration is an early occurrence in several neurodegenerative diseases. In the striatum, patients with conditions such as Huntington’s and Parkinson’s diseases (HD and PD, respectively) exhibit synapse dysfunction and loss, which strongly correlate with early symptoms of these conditions (Cheng et al., 2010; Milnerwood and Raymond, 2010; Janezic et al., 2013). However, the mechanisms contributing to synapse degeneration are not fully characterized. Importantly, it is currently unclear whether synaptic function can be fully restored in different brain regions after substantial synapse loss. Understanding these processes is crucial for developing therapeutic strategies to restore function in these neurodegenerative diseases.

The integrity of neuronal circuits in the striatum is essential for motor coordination. GABAergic medium spiny neurons (MSNs) are the principal neurons of the striatum that receive dopaminergic inputs from the substantia nigra and glutamatergic inputs from the cortex and thalamus (Gerfen, 1992; Surmeier et al., 2007; Kreitzer and Malenka, 2008). In addition, MSNs form synapses with each other and receive inhibitory inputs from local interneurons (Taverna et al., 2007, 2008; Gittis et al., 2010). There are two main populations of MSNs, D1-MSNs and D2-MSNs, that express D1 or D2 dopaminergic receptors and are part of the direct (motor facilitation) and indirect (motor suppression) pathway, respectively (Kreitzer and Malenka, 2008; Kravitz et al., 2010; Lee et al., 2016). In PD, early defects in dopaminergic inputs into the two population of MSNs are observed (Picconi et al., 2012; Schirinzi et al., 2016). In HD, synaptic dysfunction and synapse loss in MSNs appears early in the disease affecting first D2-expressing MSN pathway (Albin et al., 1992; Reiner et al., 1988). However, the molecular mechanisms that trigger these early synaptic defects are not fully understood. Understanding the mechanisms that lead to synapse damage even outside the context of PD and HD could unravel important insights into how synapses become dysfunctional.

Increasing evidence suggests that deficiency in Wnt signaling contributes to synapse dysfunction and degeneration in neurodegenerative diseases (Galli et al., 2014; Liu et al., 2014; Marzo et al., 2016; Elliott et al., 2018). For example, expression of an endogenous potent and specific secreted Wnt antagonist, Dickkopf-1 (Dkk1), is elevated in the human Alzheimer’s disease (AD) brain and in mouse models of AD (Caricasole et al., 2004; Rosi et al., 2010). Notably, amyloid-β (Aβ) oligomers, the pathogenic molecule in AD, rapidly increase Dkk1 expression as synapses degenerate (Purro et al., 2012) and knockdown or blockade of Dkk1 protects synapses against Aβ (Purro et al., 2012; Sellers et al., 2018). Moreover, inhibition of Wnt signaling in the adult mouse hippocampus by inducible expression of Dkk1 results in the loss of 40% of excitatory synapses, defects in long-term plasticity such as long-term potentiation (LTP) and long-term depression (LTD) and long-term memory (Marzo et al., 2016). In addition, in mature neurons, conditional loss of function of LRP6, a co-receptor for canonical Wnt signaling, results in synapse degeneration as mice age and exacerbates AD pathology when these animals are crossed to an AD model (Liu et al., 2014). Thus, deficiency in Wnt signaling affects the structural and functional integrity of excitatory synapses in the adult hippocampus and is linked to AD pathology. However, the contribution of dysfunctional Wnt signaling to synapse degeneration in other brain areas is less understood.

In the adult striatum, induced neuronal expression of Dkk1 in transgenic mice (iDkk1) results in a significant decrease in the number of β-catenin puncta consistent with the view that canonical Wnt signaling is compromised in iDkk1 mice (Galli et al., 2014). Notably, Dkk1 expression in the striatum also results in the specific degeneration of glutamatergic synapses from cortical but not from thalamic inputs as well as the loss of dopaminergic synapses in the absence of increased cell death (Galli et al., 2014). Importantly, Dkk1 expression triggers motor defects characteristic of striatal dysfunction (Galli et al., 2014), demonstrating a critical role of endogenous Wnt signaling in the maintenance of corticostriatal excitatory and dopaminergic synapses onto MSNs. However, the effect of Wnt deficiency on inhibitory synapses and whether these synaptic defects are reversible remain unknown.

Here, we investigated the impact of deficient Wnt signaling on the stability of GABAergic synapses in the adult striatum and whether synaptic defects are reversible. We used the iDkk1 transgenic mouse model where Dkk1 is expressed in neurons of the adult brain under a tetracycline-inducible system (Galli et al., 2014; Marzo et al., 2016). We found that induced Dkk1 expression for 14 days led to the loss of 40% of inhibitory GABAergic synapses in the adult striatum accompanied by a decrease in the frequency of miniature inhibitory currents in D2-MSNs. Thus, Dkk1 affected GABAergic synapse function on a specific subset of MSNs. Importantly, reactivation of the Wnt pathway by cessation of Dkk1 expression after substantial synapse degeneration resulted in the full recovery of GABAergic and dopaminergic synapse number. Our previous studies showed that blockade of Wnt signaling by induced expression of Dkk1 in the adult results in motor deficits (Galli et al., 2014). In the current study, we demonstrate that cessation of Dkk1 results in normal motor coordination and the ability to respond to amphetamine. These findings reveal the requirement of Wnt signaling for the maintenance of synaptic connections in the striatum and that reactivation of the Wnt pathway promotes the reassembly of functional neuronal circuits in this brain region. Thus, modulation of Wnt signaling could provide a potential therapeutic strategy to restore synaptic connectivity in neurodegenerative diseases where synapses are affected.

## Materials and Methods

### *In vivo* Induction of Dkk1 Expression in the Adult Brain

Experiments were performed according to the Animals Scientific Procedures Act United Kingdom (1986). Heterozygous tetO-Dkk1 transgenic mice (Chu et al., 2004) were crossed with heterozygous CaMKIIα-rtTA mice (Mansuy et al., 1998) to obtain the double transgenic iDkk1 (Galli et al., 2014), and their genotypes were confirmed by PCR. The triple transgenic mice iDkk1-D2R were obtained by crossing the double transgenic iDkk1 with D2R-EGFP mice (obtained from University of North Carolina MMRRC-UNC) (Gong et al., 2003). Primers used for genotyping were as follows: 5′ TGCCTTTCTCTCCACAGGTGTCC 3′ (forward) and 5′ GAGAGCACAGCGGAATGAC 3′ (reverse) for CaMKIIα-rtTA; 5′ GCGTCCTTCGGAGATGATGG 3′ (forward) and 5′ AAATGGCTGTGGTCAGAGGG 3′ (reverse) for tetO-Dkk1. In addition, we used 5′ GAGGAAGCATGCCTTGAAAA 3′ (forward) and 5′ TGGTGCAGATGAACTTCAGG 3′ (reverse) for D2R-GFP. The strain background was C57BL/6J. iDkk1 mice (or iDkk1-D2R mice) and control mice (3–6 months old) were fed with pellets supplemented with 6 mg/kg doxycycline (Dates and group) for 14 days unless otherwise indicated. Control mice were both wild-type and single transgenic animals fed with doxycycline. In some experiments, D2R-EGFP mice were used as controls. Similar numbers of male and female mice were used for cellular biology and electrophysiology experiments, whereas only males were used for behavioral experiments.

### Acute Brain Slices and Immunofluorescence Microscopy

Brains were rapidly dissected and placed into ice-cold artificial cerebrospinal fluid (ACSF) (in mM): 150 NaCl, 3 KCl, 1 CaCl_2_, 1 MgCl_2,_ 1.25 NaH_2_PO_4_, 26 NaHCO_3_, and 10 D-glucose, pH 7.4. Sagittal slices (200 μm) were cut at 4°C with a vibratome (Campden Instruments) and fixed in 4% PFA/4% sucrose (w/v) in PBS for 20–30 min at RT. Sections were subsequently washed with PBS and blocked in 10% donkey serum and 0.02% Triton X-100 in PBS for ∼4 h at RT. Primary antibodies against vesicular GABA transporter (vGAT) (Synaptic Systems, 1:1000), Gephyrin (Synaptic Systems, 1:500), GABAARα1 (Millipore, 1:500), GABABR2 (Millipore 1:250), vGlut1 (Millipore, 1:1000), PSD95 (Abcam, 1:500), D1R (Sigma, 1:200), D2R (Millipore, 1:500), Vesicular monoamine transporter 2 (VMAT2) (Acris, 1:1000), EGFP (Millipore, 1:1000), and Microtubule-associated protein 2 (MAP2) (Abcam, 1:500) were incubated overnight at 4°C. Secondary antibodies conjugated with Alexa 488, Alexa 568, and Alexa 647 from Invitrogen (dilution 1:800) were used. For the cell nucleus labeling, Hoechst (Sigma, 1:10,000) was used. Slices were washed in PBS and mounted in Fluoromount-G (SouthernBiotech). In some experiments, sagittal slices (300 μm) used for electrophysiology were stained after recordings for Neurobiotin and EGFP.

### Confocal Microscopy

Confocal images were acquired using Olympus FV1000 or Leica DMRE confocal microscopes using a 60 × 1.35 NA oil objective or 63 × 1.40 NA oil objective, respectively. Image stacks of eight equidistant planes (∼200 nm) of 76 nm/pixel × 76 nm/pixel were taken for each field. Analyses were performed using Volocity software (Perkin Elmer). Images were acquired from four to six mice. Three to four brain slices were evaluated per mouse, and three images were taken per slice. Synaptic puncta volume and number as well as VMAT2 intensity were quantified in Volocity using custom protocols based on standard thresholding techniques. Number of pre- or postsynaptic puncta and number of synapses (determined by the co-localization of a pre- and postsynaptic marker) were normalized to control mice that were fed with doxycycline for 14 days and expressed as percentage of control. Data from different independent experiments were pooled and significance was tested by non-parametric Mann–Whitney or Kruskal–Wallis ANOVA depending on the number of comparisons.

### Accelerating Rotarod

Mice fed for 14 days with doxycycline and subsequently fed with normal diet for additional 2–3 weeks were evaluated on an accelerating rotarod task. Mice were placed on a rotarod (Med Associates) accelerating from 4 to 40 rpm in 5 min and the latency to fall was recorded. Mice performed five consecutive trials per day (∼30 s rest between trials), for a total of four consecutive days. Maximum trial length was 5 min, after which the animals were returned to the cage. Data from eight control and eight iDkk1 mice was evaluated by repeated measures one-way ANOVA.

### Amphetamine Induced Locomotion

Motor activity was recorded in eight identical activity monitor chambers (43 cm × 43 cm) equipped with 16 infrared light emitters and detectors (Med Associates), connected to a computer that counts the number of times the photo beams are broken (as the animal crosses between the emitter and the detector). The total number of horizontal beam breaks was used as a measure of locomotion. Mice fed with doxycycline for 14 days and subsequently returned to normal diet for an additional 2–3 weeks were placed individually in the activity chamber and habituated for 1 h prior to the experiment. After habituation, mice were intraperitoneally administered with amphetamine (D-amphetamine sulfate, Sigma, 2 mg/kg in saline) or saline as control. Locomotion was monitored for 1 h immediately after the injection by registering the infrared photo beam interruptions. Significance was evaluated by two-way ANOVA (four control mice injected with saline, five control mice injected with amphetamine, four iDkk1 injected with saline, and four with amphetamine).

### Slice Preparation for Electrophysiology

Brains were rapidly removed and placed into ice-cold “slicing solution” containing (in mM): 75 sucrose, 87 NaCl, 25 NaHCO_3_, 2.5 KCl, 1.25 NaH_2_PO_4_, 10 glucose, 0.5 CaCl_2_, and 7 MgCl_2_ bubbled with 95% O_2_ and 5% CO_2_. Sagittal slices (300 μm) containing the striatal region were prepared with a vibratome (Leica, Germany) and kept in an oxygenated holding chamber containing (in mM): 125 NaCl, 26 NaHCO_3_, 2.5 KCl, 1.26 NaH_2_PO_4_, 25 glucose, 2 CaCl_2_, and 1 MgCl_2_ (Krebs solution) at RT and maintained at pH 7.4 by permanent bubbling with 95% O_2_ and 5% CO_2_.

### Electrophysiological Recordings

After 1-h recovery period, slices were transferred to a recording chamber on an upright microscope with Normanski-differential interference contrast optics (Zeiss Axioskop, Germany) and continuously perfused with oxygenated Krebs solution at RT (22–24°C). Patch-clamp recording pipettes were made from thick-walled borosilicate glass (GC150F, Harvard Apparatus, Kent, United Kingdom) using a vertical pipette puller (Narashige) and filled with a Cs Gluconate-based pipette solution, containing (in mM): 139 D-gluconic acid lactone, 10 HEPES, 10 EGTA, 10 NaCl, 0.5 CaCl_2_, 1 MgCl_2_, 1 ATP, and 0.5 GTP adjusted to pH 7.2 with CsOH and 3 μg/ml Neurobiotin (Vector Labs) or 135 K-gluconate, 10 HEPES, 0.1 EGTA, 10 KCl, 0.5 CaCl_2_, 2 MgCl_2_, 5 phosphocreatine, 2 ATP, and 0.5 GTP. Individual MSNs from the dorsal part of the striatum were voltage-clamped at −80 mV following establishment of whole-cell mode. Membrane capacitance was estimated “online” using the Axopatch 200B amplifier capacitance cancelation circuit to cancel the current capacitance transients evoked in voltage-clamp mode in response to 5-mV, 20-ms duration depolarizing voltage steps evoked at −70 mV as previously described (Sherman-Gold, 1993).

Neurons were identified by morphological and electrophysiological criteria (cell size, membrane capacitance, and cell response to depolarizing current injection). In triple transgenic mice, iDkk1-D2R-GFP (which labels MSNs from the indirect pathway), neurons were identified by the expression of EGFP. Some large cholinergic interneurons express D2R; therefore, GFP-positive cells that had a morphology (cell body diameter of more than 20 μm) and a firing pattern (accommodating spike firing) typical of large cholinergic interneurons were excluded from the mEPSCs and the morphology analyses. In these experiments, Cs-gluconate-based internal solution was used. Currents were recorded using an Axopatch 200B Amplifier (Axon Instruments, United States), filtered at 1 kHz, and digitized in the computer at 10 kHz with a Micro 1401 interface (Cambridge Electronic Design, United Kingdom). The data were acquired with Win EDR and analyzed using Win EDR and Win WCP (Strathclyde Electrophysiology Software freely available at: http://spider.science.strath.ac.uk/sipbs/showPage.php?page=software_ses).

Whole-cell current-clamp recordings were made with patch pipettes containing K+-gluconate-based pipette solution. Once whole-cell configuration was stabilized, the holding current was adjusted to set a membrane potential of −70 mV and then 500-ms duration current steps were applied at 10-s intervals from 10 to 100 pA in amplitude in 10-pA increments, and the resulting membrane potential depolarizations and action potential firing were recorded at a bandwidth of 2 kHz and digitized at 10 kHz. To record the miniature postsynaptic currents, 0.5 μM Tetrodotoxin (TTX) was added to the Krebs solution. Recordings of mIPSCs were made at +10 mV. The mIPSC decay was best fit by two exponential components using the following equation where current (*I*) is measured in pA and time constant τ in ms: *I*_*total*_ = *I*_fast_exp(–time/τ_fast_) + *I*_slow_exp(–time/τ_slow_), where τ_fast_ and τ_slow_ are the deactivation time constants for the fast and slow components, respectively. To summarize, in the mIPSC deactivation kinetics, a weighted decay time constant was calculated using the following equation: τ_weighted_ = (τ_fast_*I*_fast_ + τ_slow_*I*_slow_)/(*I*_fast_ + *I*_slow_).

Data are presented as mean ± standard error (SE), with *n* indicating the number of cells, which also corresponds to the number of slices since, for each cell, excitability was first recorded without TTX and then TTX was added to the external solution to record mIPSCs. Statistical analyses were performed using SPSS 26. A log10 transformation of the mIPSC frequency values was applied to meet parametric test criteria. Two-way ANOVA (genotype and type of neuron) was performed and revealed a significant difference between different types of neurons (D1R and D2R MSN; *p* = 0.0001) and a significant interaction between the genotype and the type of neurons (*p* = 0.006). Student’s *t*-test was then used to compare pairs of observations within each type of neuron patched in whole-cell patch-clamp configuration.

### Filling, Staining, and Reconstruction of MSN Morphology

To track the dendritic arbor of the recorded neurons, 3 μg/ml Neurobiotin (Vector Labs) was added into the intracellular solution. The MSNs were patched in whole-cell clamp configuration for at least 15 min to allow the spread of the Neurobiotin. Consequently, the slices were fixed in 4% PFA for 24–48 h, washed in 0.1 M PBS, blocked in 10% donkey serum, permeabilized with 0.05% Triton X-100, and then incubated for 2 h with 2 mg/ml Alexa Fluor^®^ 594 Streptavidin (Thermo Fisher Scientific). For the neuronal reconstruction, the open-source software ImageJ (Fiji) (Schindelin et al., 2012) was used.

### Statistical Analyses

All data were explored for normality, outliers, and fulfillment of statistical test assumptions in SPSS 26 (IBM Corp., Armonk, NY, United States). When samples are normally distributed and their variance are homogeneous, parametric tests were used. Student’s *t*-test to compare pairs of observations and one-way ANOVA or two-way ANOVA with Tukey’s *post hoc* correction for multiple group comparisons were made. When data failed to meet those criteria, we used a non-parametric test such as Mann–Whitney *U* to compare two groups or Kruskal–Wallis non-parametric ANOVA. Generally, *p < 0.05, **p < 0.01, and ***p < 0.005.

## Results

### Induced Dkk1 Expression Triggers GABAergic Synapse Degeneration in the Adult Striatum

**FIGURE 1:**
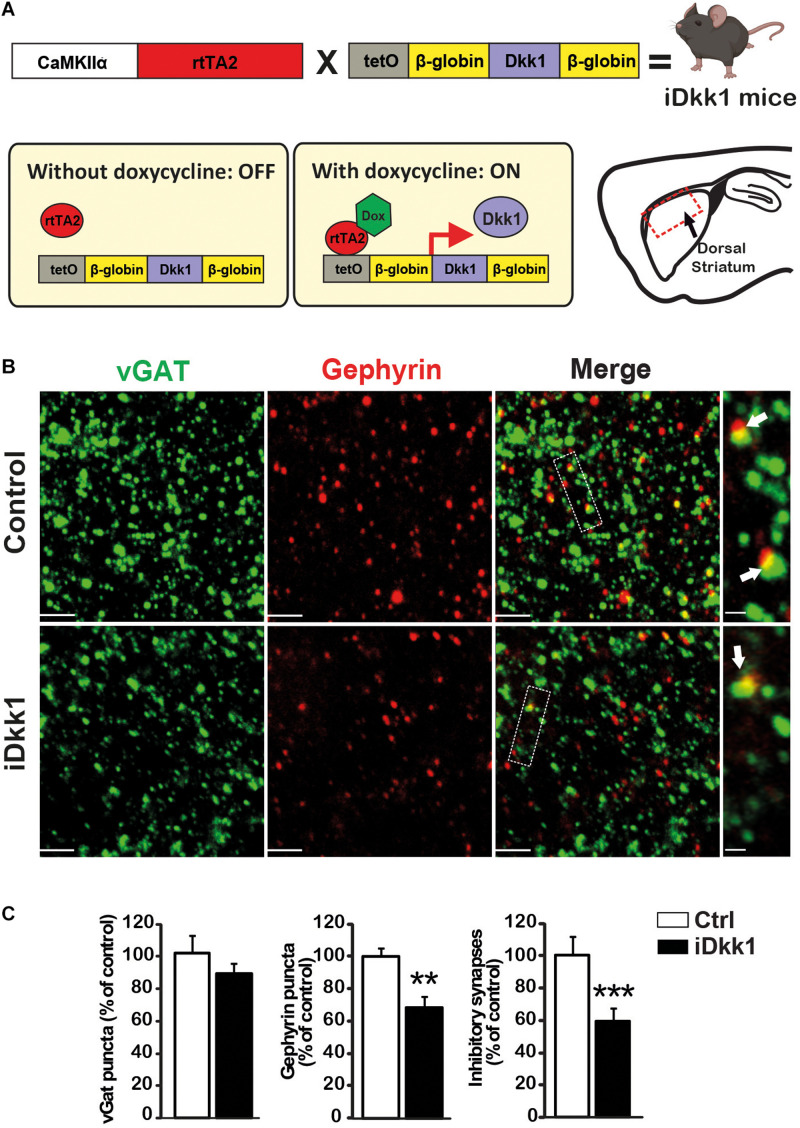
Induction of Dkk1 induces 40% loss of inhibitory synapses in dorsal striatum. **(A)** Schematic representation of the generation of iDkk1 mice by the crossing of mice carrying the Dkk1 cDNA under the control of a doxycycline responsive promoter (tetO) with mice carrying the doxycycline-controlled transactivator 2 (rtTA2) downstream of the CaMKIIα promoter. In the absence of doxycycline (OFF), Dkk1 expression was not induced, whereas in animals fed with doxycycline (ON), Dkk1 is expressed, notably but not exclusively, in the dorsal striatum, the region under study here (red square on the sagittal brain scheme) in iDkk1 mice. **(B)** Confocal images from dorsal striatum show a dramatic reduction in inhibitory synapse number (white arrows) based on the co-localized pre- and postsynaptic markers; vGAT (green) and Gephyrin (red) puncta. The scale bars represent 2 μm; Dashed boxes correspond to enlarged areas depicted on the right-most panels, scale bar: 0.5 μm. **(C)** Forty percent of inhibitory synapses were lost in iDkk1 mice as shown on the quantification of pre- and postsynaptic markers and their co-localization. *N* = 5 mice per group; ^∗∗^*p* < 0.01; ^∗∗∗^*p* < 0.005, Mann–Whitney.

Our previous studies showed that deficient Wnt signaling triggers the loss of excitatory and dopaminergic synapses in the adult mouse striatum (Galli et al., 2014). We therefore examined whether GABAergic synapses, which are the most abundant synapses in the striatum, were also affected by a deficiency in the Wnt pathway. We used the double transgenic iDkk1 mouse model in which Wnt signaling activation, in response to Wnts, is blocked by inducing the expression of the specific and potent Wnt antagonist Dkk1 (Galli et al., 2014; Marzo et al., 2016; Figure 1A). We used a transgenic mouse line expressing Dkk1 under the control of the tetracycline-inducible system and the Ca^2+^/calmodulin-dependent protein kinase II (CaMKII) promoter resulting in the expression of the transgene in principal neurons of brain (Mansuy et al., 1998). Previous studies showed that expression of the LacZ gene under this promoter system is mainly expressed in large neurons in the striatum but not in small neurons, suggesting that the CamKII promoter probably drives expression in MSNs and in large cholinergic interneurons but not in small interneurons (Odeh et al., 2011). Our previous studies showed that Dkk1 is expressed in the striatum after adult mice were fed with doxycycline (Galli et al., 2014). Using this inducible system, Wnt signaling was unaffected during embryonic and postnatal development when this pathway is required for embryonic patterning, axon guidance, and synapse formation (Budnik and Salinas, 2011; Nusse and Clevers, 2017). In addition, our previous studies showed that induction of Dkk1 expression for 14 days does not affect cell death in the striatum (Galli et al., 2014). To determine the impact of Dkk1 on GABAergic synapses, we evaluated the co-localization of the inhibitory presynaptic marker vGAT and the postsynaptic marker Gephyrin in the dorsal striatum. Single transgenic mice that do not express Dkk1 were fed with doxycycline for 14 days and used as controls. In double transgenic mice, induction of Dkk1 expression did not affect the number or volume of presynaptic terminals labeled with vGAT (Figures 1B,C and Supplementary Figure 1A) but induced a significant decrease (30–35%) in the number of Gephyrin puncta. Importantly, Dkk1 induced a significant decrease (40%) in the number of inhibitory synapses based on the co-localization of vGAT and Gephyrin puncta (Figures 1B,C). However, Dkk1 did not affect the volume of the postsynaptic scaffolding protein Gephyrin (Supplementary Figure 1B). Next, we investigated whether the number of GABAA receptors (GABAAR α1 and GABABR2) was affected by Dkk1 induction. GABAAR α1 cluster number was unaffected by Dkk1 as assessed by immunostaining (Supplementary Figure 1C). In contrast, GABABR2 cluster number and co-localization with VAMP2, a presynaptic marker, were significantly decreased in the striatum of iDkk1 mice (Supplementary Figure 1D). Thus, blockade of Wnt signaling in the adult striatum results in the loss of GABAergic synapses and a decrease in GABABR2 puncta, demonstrating a critical role for endogenous Wnts in the maintenance of inhibitory synapses.

### Dkk1 Affects GABAergic Synapses of the Indirect Pathway in the Striatum

**FIGURE 2:**
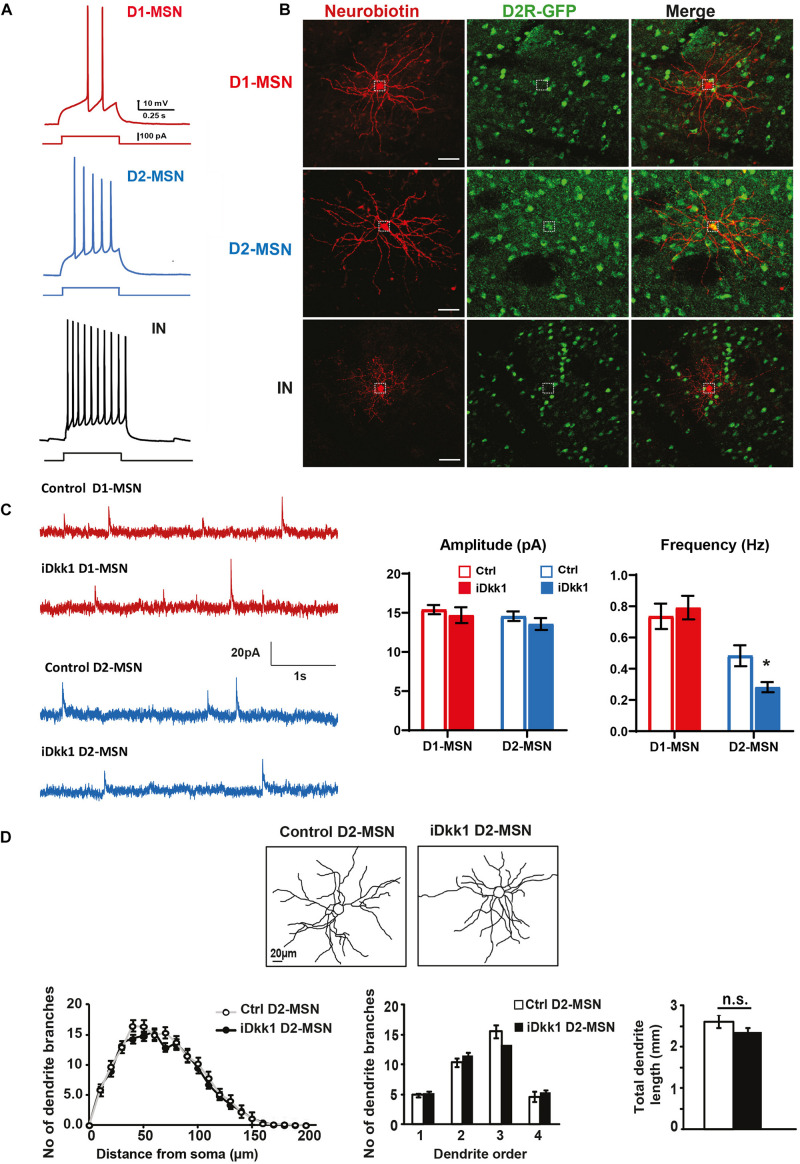
Dkk1 decreases the frequency of mIPSCs only in D2-MSNs, without affecting their morphology. **(A)** Resting membrane potential (Vm) was similar in all conditions. Holding currents were adjusted to set a membrane potential of −70 mV, and these recordings were thus performed at rest. Representative traces showing action potentials (AP) elicited with a 500-ms current step of 100 pA. D1-MSNs (red) show a low firing rate. In contrast, D2-MSNs (blue) and interneurons (black, IN) are highly excitable. IN and D1-MSNs were distinguished based on their morphology and their AP firing patterns. **(B)** Confocal images of Neurobiotin-filled neurons in D2-iDkk1 mice. EGFP-labeled D2-MSNs were filled with Neurobiotin and show a large dendritic arbor. EGFP-negative neurons reveal either extensive branching (D1-MSNs) or small dendritic arbors (INs). The dashed circles represent a neuronal cell body. The scale bar represents 40 μm. **(C)** Representative traces of mIPSCs from D1-MSN (top traces) and D2-MSNs (lower traces) as indicated from control mice and from iDkk1 mice after induction of Dkk1 expression. mIPSC amplitude was not affected by Dkk1 in D1-MSNs or D2-MSNs (top panel). In contrast, the frequency of mIPSC is reduced only in D2-MSNs after induction of Dkk1 but not in D1-MSNs (lower panel). *N* = 17–21 cells recorded from four animals per condition; ^∗^*p* < 0.05, two-way ANOVA with interaction followed by Tukey’s *post hoc* tests. **(D)** Reconstruction of Neurobiotin-filled D2-MSNs (left panels). Sholl analysis (number of intersections), quantification of dendritic branches per dendritic order (1st, 2nd, 3rd, and 4th order) (repeated measure one-way ANOVA), and total length of dendrites (*t*-test) showed that Dkk1 does not affect D2-MSN dendritic morphology. *N* = 15 cells were evaluated from seven animals of each genotype.

Inhibitory synaptic connections in the striatum come from reciprocal contacts between MSNs and local interneurons (Koos and Tepper, 1999; Koos et al., 2004; Taverna et al., 2008). As MSNs expressing D1R (D1-MSNs) or D2R (D2-MSNs) are characteristic of the direct and indirect basal ganglia pathways, respectively, we decided to determine whether Dkk1 specifically affects a subpopulation of MSNs. We therefore crossed iDkk1 mice with D2R-BAC EGFP mice, which specifically express EGFP in D2-MSNs (Gong et al., 2003; Wang et al., 2006). We verified that EGFP-labeled neurons were D2-MSNs by examining their action potential firing profile (Figure 2A) coupled to *post hoc* EGFP immunolabeling. Our electrophysiological recordings revealed a clear difference in the pattern of firing between D1-MSNs (low excitability) and other types of neurons (D2-MSNs and interneurons). For example, D2R-EGFP cells exhibited sustained and high excitability when compared to EGFP-negative neurons possibly representing D1-MSNs (Figure 2A and Supplementary Figure 2) as described previously (Gertler et al., 2008; Cazorla et al., 2012). Dkk1 expression was induced in the adult brain by feeding D2-iDkk1 mice with doxycycline for 14 days. Single transgenic D2 mice that do not express Dkk1 were also fed with doxycycline for 14 days and used as controls. No significant differences of resting membrane potential (zero current potential) were observed between D1- and D2-MSNs or following Dkk1 expression (control D1-MSN, −73.7 ± 1.53 mV; iDkk1 D1-MSN, −75.0 ± 0.97 mV; control D2-MSN, −75.8 ± 1.49 mV; iDkk1 D2-MSN, −75.8 ± 0.88 mV). These data were in accordance with analyses of electrical properties of the cells, which showed no significant difference in estimated membrane capacitance (control D1-MSN, 25.9 ± 1.76 pF; iDkk1 D1-MSN, 23.7 ± 0.98 pF; control D2-MSN, 25.4 ± 1.15 pF; iDkk1 D2-MSN, 22.0 ± 0.62 pF). Recorded cells were then filled with Neurobiotin to visualize their dendritic arborization to allow quantification of their size and complexity (Figure 2B).

Once we had established our ability to identify and record from different neurons in the striatum, we next investigated possible functional changes in inhibitory synaptic function by recording miniature postsynaptic currents (mIPSCs) in MSNs when Dkk1 was induced. mIPSCs were recorded on D2-MSNs (GFP labeled) and D1-MSNs (GFP-negative cells, *post hoc* labeled with Neurobiotin and assessed for morphology). We found that Dkk1 did not affect the amplitude of the mIPSCs in either population (Figure 2C, upper panel). In contrast, a significant decrease in the frequency of mIPSCs (∼50%) was observed in D2-MSNs (GFP labeled) whereas no changes were recorded from D1-MSNs (Figure 2C). For both classes of neurons, Dkk1 expression did not affect mIPSC current kinetics (weighted tau decay: D1-MSN, 18.5 ± 0.91 ms; control D2-MSN, 20.1 ± 0.99 ms; iDkk1 D1-MSN, 20.1 ± 1.00 ms; iDkk1 D2-MSN, 19.3 ± 0.67 ms). These results reveal that deficiency in Wnt signaling affected inhibitory synaptic transmission in a subpopulation of neurons in the dorsal striatum, the D2-MSNs.

We next interrogated the possibility that Dkk1 affects the frequency of mIPSCs in the D2R-neurons by affecting dendritic arborizations. The morphology of recorded D2R-GFP cells in control and Dkk1 expressing mice was quantified by Sholl analyses. However, no significant differences were observed in dendritic arborization (number of dendritic branches) or in the total dendritic length (Figure 2D), suggesting that Dkk1 affected the frequency of mIPSCs in D2-MSN neurons without inducing without inducing changes in dendritic arborization. We found that the membrane capacitance was the same in the presence or absence of Dkk1. Previous studies have shown that dendritic morphology is correlated with changes in membrane capacitance (Matsumura et al., 2018). These results are consistent with our finding that Dkk1 does not affect dendritic morphology.

### The Loss of Inhibitory Synapses Is Reversible

**FIGURE 3:**
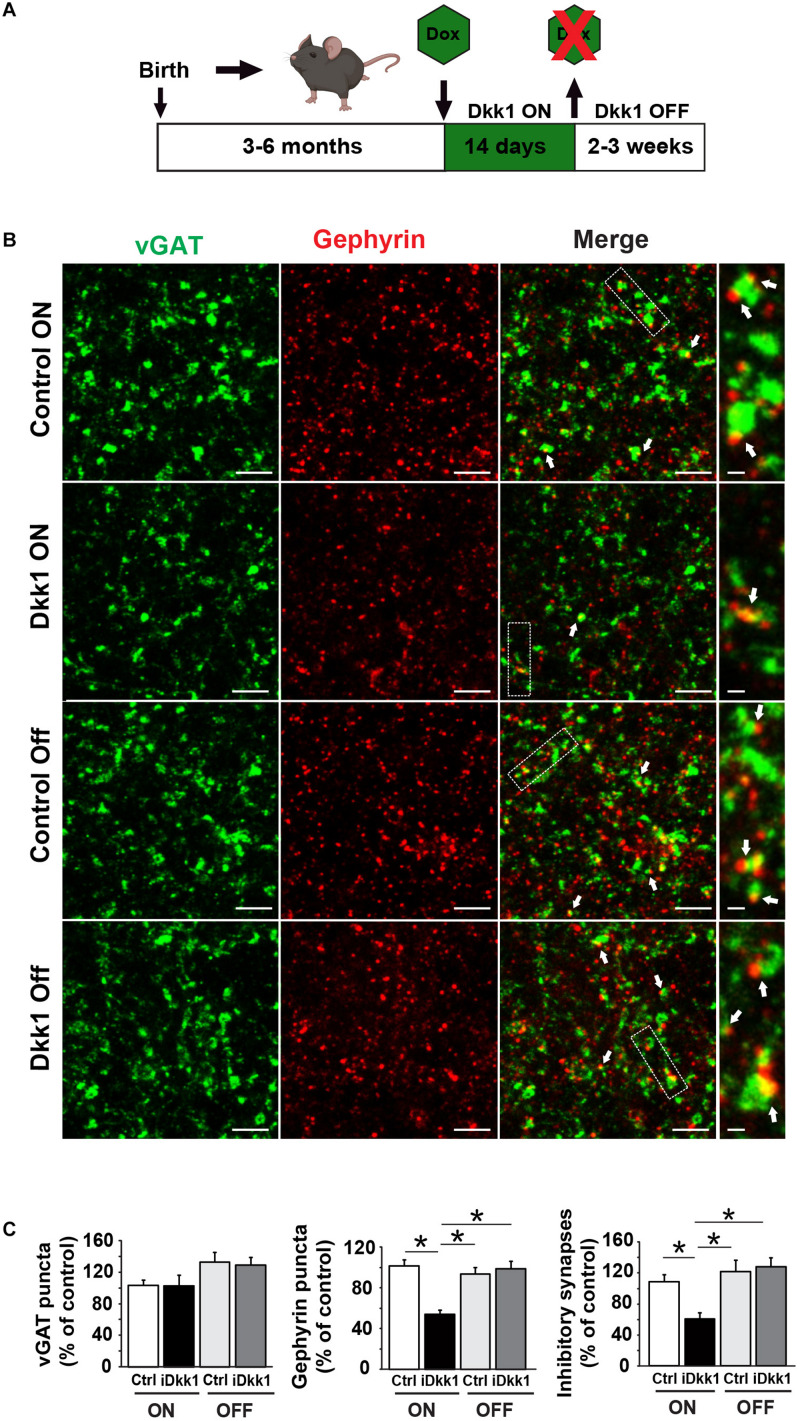
Cessation of Dkk1 expression leads to full recovery of inhibitory synapse number. **(A)** Schematic diagram of the iDkk1 ON–OFF model. Three- to six-month adult mice were fed with doxycycline food (green hexagon) for 14 days, which induced the expression of Dkk1. Doxycycline feeding, and therefore induced Dkk1 expression, was then stopped for 2–3 weeks before using the animals for experiments. **(B)** Confocal images show a reduction in the number of inhibitory synapses based on the co-localization (white arrows) of pre-synaptic vGAT (green) and postsynaptic Gephyrin (red) puncta after induction of Dkk1 following doxycycline (iDkk1 ON) when compared to control mice exposed to doxycycline (Control ON). Synapse number recovered after cessation of Dkk1 expression (iDkk1 OFF). The scale bars represent 2 μm; Dashed boxes correspond to enlarged areas depicted on the right-most panels and scale bars represent 0.3 μm. **(C)** Quantification of the number of synapses based on the co-localization of pre- and postsynaptic markers. ^∗^*p* < 0.05, Kruskal–Wallis.

It is well documented that in several neurodegenerative diseases, synapses are lost at early stages (Scheff et al., 2006; Shankar and Walsh, 2009; Milnerwood and Raymond, 2010; Soukup et al., 2018), but it remains poorly understood if synapse number and function can be fully restored after substantial synapse degeneration. We recently found that excitatory synapse loss in the hippocampus triggered by induced Dkk1 expression can be fully reversed by reactivation of Wnt signaling (Marzo et al., 2016). These findings led us to investigate whether turning off Dkk1 expression could restore the number of inhibitory synapse to that of control levels. For these experiments, we induced Dkk1 expression for 14 days using doxycycline (ON period) followed by the withdrawn of doxycycline, for 2 weeks (iDkk1 ON–OFF) (Figure 3A), as previously done in the hippocampus (Marzo et al., 2016). As shown in Figure 1, induction of Dkk1 expression for 2 weeks did not affect the number of vGAT presynaptic terminals (Figures 3B,C) but significantly reduced the number of postsynaptic Gephyrin puncta and therefore the number of GABAergic synapses, as measured by the co-localization of vGAT and Gephyrin (Figures 3B,C). Upon cessation of Dkk1 expression for 2 weeks, the numbers of Gephyrin puncta and inhibitory synapses were restored to control levels (Figures 3B,C). These results demonstrate that inhibitory synapse number recovers after restoring Wnt signaling in the adult striatum.

### Decreased Dopaminergic Synapse Number Is Reversible Upon Dkk1 Termination

**FIGURE 4:**
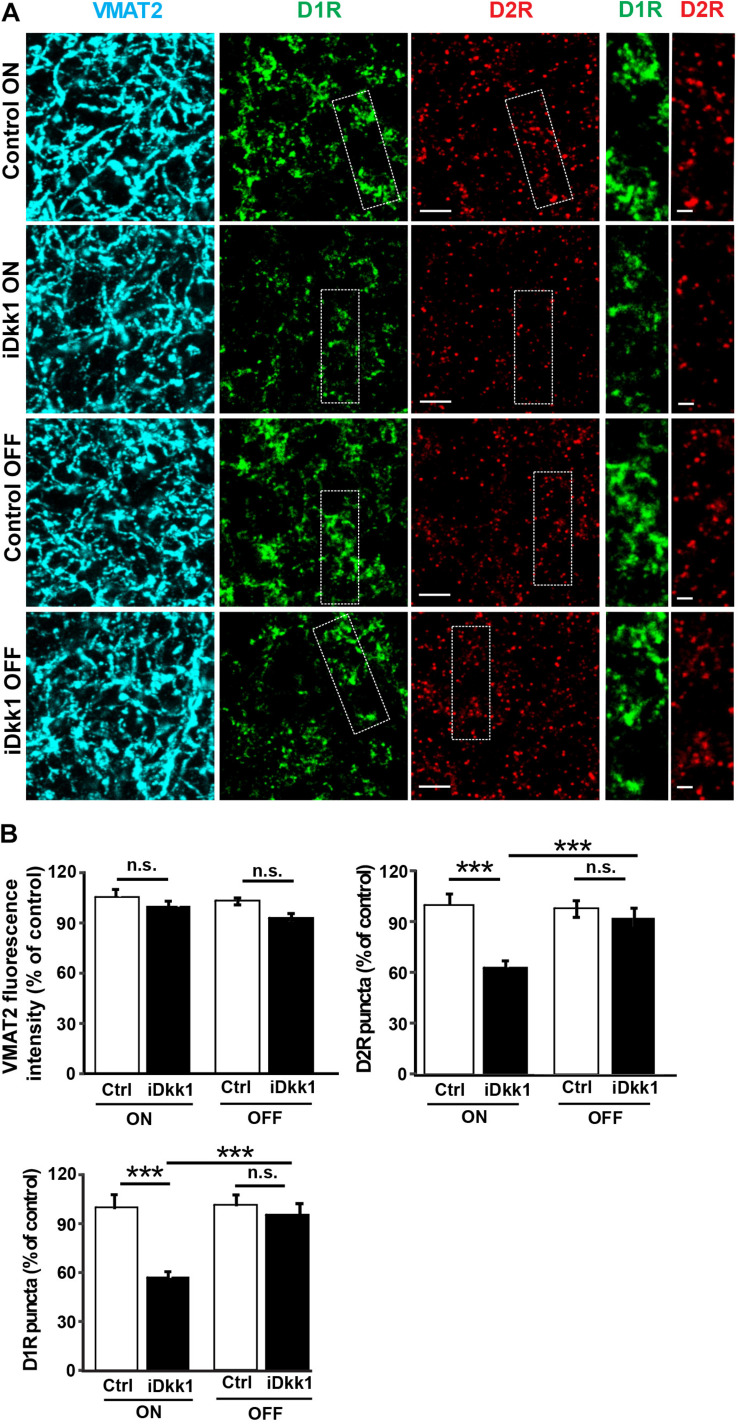
Dopaminergic receptor cluster number is restored when Dkk1 expression is turned off. **(A)** Confocal images from the adult striatum reveal no change in dopaminergic afferents labeled with VMAT2 (blue) following induction of Dkk1 (iDkk1 ON) when compared to control mice exposed to doxycycline (Control ON). In contrast, the numbers of D1R (green)- and D2R-labeled puncta (red) decreased in the presence of Dkk1 (iDkk1 ON) but were restored with cessation of Dkk1 expression (iDkk1 OFF). The scale bars represent 2.5 μm; dashed squares are enlarged on the side and the scale bars represent 0.5 μm. **(B)** Quantification of the VMAT2 intensity and D1R- and D2R-labeled puncta number relative to the control show that the number of D1R- and D2R-labeled puncta fully recovered after turning off Dkk1 expression. *N* = 5 mice per group; ^∗∗∗^*p* < 0.001, Kruskal–Wallis.

A striking defect induced by Dkk1 in the dorsal striatum is the loss of dopaminergic receptors (Galli et al., 2014). We next examined whether withdrawal of Dkk1 after synapse degeneration affects the number of D1R and D2R receptor clusters. We first induced Dkk1 expression for 2 weeks (ON) in the striatum of iDkk1 mice and evaluated endogenous D1R and D2R receptor clusters and dopaminergic axon terminals labeled with an antibody against the vesicular monoamine transporter 2 (VMAT2). Dkk1 did not induce axon terminal degeneration as determined by VMAT2-labeled processes (Figure 4A) as we previously showed (Galli et al., 2014). In contrast, Dkk1 expression triggered a 40% loss in D1R- and D2R-labeled puncta (Figures 4A,B). Conversely, termination of Dkk1 expression resulted in the full recovery of the number of D1R and D2R receptor clusters to control levels (Figures 4A,B). Together, these studies showed that deficient Wnt signaling triggers the loss of dopaminergic and GABAergic synapses in the striatum but cessation of Dkk1 expression results in the full regeneration of these synapses.

### D1R Recovery Correlates With Normal Amphetamine-Induced Locomotion After Dkk1 Cessation

**FIGURE 5:**
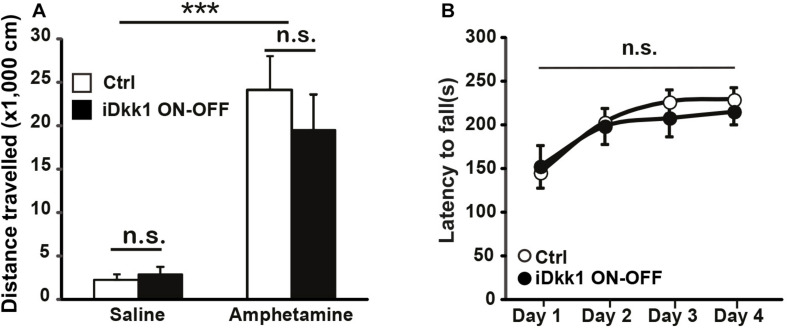
Behavioral defects are reversed by cessation of Dkk1 expression. **(A)** Intraperitoneal injection of amphetamine (2 mg/kg) increases the traveled distance in the open field; ^∗∗∗^*p* < 0.001, two-way ANOVA. However, no differences were observed between control and iDkk1 ON–OFF mice (*p* > 0.05) by two-way ANOVA. *N* = 4–5 mice per group. **(B)** Performances on the accelerating rotarod are identical between control and iDkk1 mice following cessation of Dkk1 expression (iDkk1 ON–OFF); *p* > 0.05, repeated measures one-way ANOVA. *N* = 8 mice per group.

Dopamine innervation to the dorsal striatum is essential for motor control. Potentiation of dopamine signaling by psychostimulants like amphetamine increases locomotion through D1Rs (Xu et al., 1994; Natarajan and Yamamoto, 2011). Indeed, the decrease in D1R and D2R expression in the striatum upon induction of Dkk1 for 2 weeks correlates with the inability of amphetamine to increase locomotion in iDkk1 mice (Galli et al., 2014). As termination of Dkk1 expression induces the recovery in the number of D1R clusters (Figure 4), we predicted that iDkk1 mice that had been taken off doxycycline feeding would respond normally to amphetamine. Indeed, amphetamine exposure significantly increased locomotion in control mice. The iDkk1 ON–OFF mice (Figure 3A) responded to amphetamine in a similar manner to control mice injected with amphetamine (Figure 5A). Given our previous finding that induction of Dkk1 results in the lack of response to amphetamine (Galli et al., 2014), our results here suggest that the response to the psychostimulant was fully restored following the cessation of Dkk1 expression.

### Restoration of Wnt Signaling Leads to Normal Motor Coordination

We have previously shown that induced expression of Dkk1 in the adult brain altered normal striatum-regulated behavior, such as motor coordination and learning, evaluated by the performance of animals on the accelerating rotarod (Galli et al., 2014). Although the accelerating rotarod tests striatal function (Dang et al., 2006; Yin et al., 2009), it also assesses cerebellar function (Shiotsuki et al., 2010; Woo et al., 2018). As Dkk1 expression is driven by the CaMKII promoter (Figure 1), which is not expressed in the adult cerebellum (Mansuy et al., 1998), the defects in the rotarod are mainly due to the impact of Dkk1 on striatal function. The defect in the rotarod exhibited by iDkk1 mice is probably directly linked to loss of dopaminergic receptor clusters. Given the recovery of synapse number and the response to amphetamine in iDkk1 mice following cessation of Dkk1 expression, we decided to test the latency to fall in these animals when placed on the accelerating rotarod. We observed that upon termination of Dkk1 expression, iDkk1 mice performed well on the rotarod during the first day, when animals were naïve to the task, and also throughout days 2–4 in a similar manner to control animals (Figure 5B). Both iDkk1 and control mice exhibited similar improvement between sessions (Figure 5B), suggesting that both sets of animals have the same ability to learn. Thus, cessation of Dkk1 expression following synapse degeneration results in the full recovery of striatum function.

## Discussion

We previously showed that blockade of endogenous Wnts through the inducible expression of Dkk1 results in the substantial loss of excitatory and dopaminergic synapses in the adult striatum without affecting cell viability (Galli et al., 2014). In the current study, we demonstrate that Wnt signaling also plays an important role in inhibitory synapse integrity in the adult striatum. Indeed, we show for the first time that Dkk1 affects the function of D2-MSN GABAergic synapses. Importantly, reactivation of the Wnt signaling pathway after significant synapse loss results in the normal number of GABAergic and dopaminergic synapses, normal motor coordination and learning, and response to amphetamine regarding motor activity.

Studies have demonstrated that Wnt signaling is required for synapse integrity in several areas of the adult brain. Induced expression of Dkk1 in the adult hippocampus triggers the loss of 40% of excitatory synapses. However, inhibitory synapses are unaffected, suggesting that Dkk1 does not target inhibitory synapses in the hippocampus (Marzo et al., 2016). In the striatum, in contrast, induced Dkk1 expression resulted in a significant decrease in the number of inhibitory synapses. Importantly, patch-clamp recordings revealed that Dkk1 expression significantly decreased the frequency of mIPSCs in D2R-expressing MSNs, but it does not seem to affect GFP-negative neurons representing mostly D1R-expressing MSNs. D2-MSNs are part of the indirect pathway, which is one of the two main outputs of the striatum and is often described as the “no go” pathway as it inhibits thalamo-cortical activation (Bamford et al., 2018). *In vivo* optogenetic excitation of indirect-pathway MSNs in mice leads to a parkinsonian state, characterized by increased freezing, bradykinesia, and decreased locomotor activity (Kravitz et al., 2010). Thus, our finding that Dkk1 reduced inhibitory transmission in D2-MSN neurons suggests a higher activity in these neurons. However, we previously reported that 50% of excitatory corticostriatal synapses are lost after Dkk1 induction (Galli et al., 2014), which could balance for the loss of inhibitory inputs onto MSNs of the indirect pathway. Thus, further studies are required to determine the effect of Dkk1 on the direct or indirect pathway at the network level.

What are the mechanisms underlying the difference in the susceptibility of inhibitory synapses in the hippocampus and the striatum and also between D1-MSNs and D2-MSNs? Dkk1 antagonizes Wnt signaling by binding to the Wnt co-receptor, the low-density lipoprotein receptor-related protein 6 (LRP6), therefore preventing the formation of the Wnt–Fz–LRP6 complex and the subsequent activation of the Wnt signaling cascade (Mao et al., 2001; Buechler and Salinas, 2018). Given that inhibitory synapses differ between brain regions and between neuronal types (Kubota et al., 2016), it is possible that inhibitory synapses in the hippocampus and striatum, or between D1-MSNs and D2-MSNs, express a different level of LRP6 or other Wnt receptors at the cell surface, making some of these synapses more susceptible to Dkk1 than others. Further studies are required to determine the mechanisms that lead to this different susceptibility to Dkk1.

Deficiency in Wnt signaling has been linked to neurodegenerative diseases. In particular, increasing evidence suggests that deficiency in canonical Wnt signaling could contribute to synapse degeneration in Alzheimer’s disease (Purro et al., 2012; Liu et al., 2014; Marzo et al., 2016). However, links between Wnt signaling and PD/HD are beginning to emerge. First, proteomic analyses of dopamine neurons from the rotenone-induced PD model reveals deficits in Wnt signaling (Stephano et al., 2018). Second, gain of function of Wnt4 is protective in a model of PD in the fly (Wu et al., 2019). Third, several genes linked to PD have been shown to interact with Wnt components or modulate Wnt signaling (Rawal et al., 2009; Berwick and Harvey, 2014). Of particular interest is the leucine-rich repeat kinase 2 (LRRK2), a serine/threonine kinase linked to familial PD, which interacts with several components of the Wnt pathway such as LRP6 and Dishevelled (Dvl) (Sancho et al., 2009; Berwick and Harvey, 2012). Importantly, familial LRRK2 mutations exhibit decreased Wnt signaling probably due to defects in the interaction with Wnt components (Berwick and Harvey, 2012). Furthermore, the vacuolar protein sorting protein 35 (Vps35), an essential retromer subunit, regulates Wnt secretion (Belenkaya et al., 2008) and has been linked to autosomal dominant late-onset PD (Zimprich et al., 2011). Finally, polymorphisms in certain Wnt signaling components including Gsk3β have been linked to PD (Berwick and Harvey, 2014). However, a link between Wnt signaling and HD is less clear. For example, a mutant huntingtin has been linked to β-catenin degradation (Godin et al., 2010) and HD Research Crossroad database showed Wnt signaling as a possible target in HD (Kalathur et al., 2012). Our finding that Wnt deficiency affects inhibitory synapses in D2-MSNs is interesting because these neurons are particularly affected in dopamine depletion PD models (Day et al., 2006) and are vulnerable in human HD and in mouse models of HD (Albin et al., 1992; Andre et al., 2011a,b; Cepeda et al., 2013). Moreover, deficient Wnt signaling in the striatum triggers synapse degeneration and motor defects (Galli et al., 2014) as observed in PD and HD. Together, these findings suggest a potential link between dysfunction of Wnt signaling and PD and HD.

Although a link between Wnt signaling in PD and HD has not been established, our studies demonstrate that deficiency in Wnt signaling leads to the loss of excitatory, dopaminergic, and inhibitory synapses in the striatum (Galli et al., 2014). Notably, our results show that activation of the Wnt pathway by withdrawal of Dkk1 expression in iDkk1 mice after substantial synapse degeneration results in the restoration of inhibitory and dopaminergic synapse number to control levels. Consistently with recovery of synapse number, motor learning and coordination as well as the response to amphetamine were normal after Dkk1 cessation. Thus, reactivation of the Wnt pathway could be used as an approach to restore whole circuit function in the adult striatum even after significant functional defects.

This study provides new insights into the role of Wnt signaling in the maintenance of synaptic connections in the striatum. Induced Dkk1 expression triggers the degeneration of 40% of inhibitory GABAergic synapses in the adult striatum. However, electrophysiological recordings revealed that Dkk1 affects GABAergic synapse function on D2R-MSNs. Importantly, cessation of Dkk1 expression after substantial synapse degeneration in the adult striatum results in the recovery of inhibitory and dopaminergic synapses, motor coordination, and the ability to respond to amphetamine. Thus, reactivation of the Wnt signaling pathway promotes the restoration of functional neuronal circuits in the adult striatum. These findings suggest the exciting possibility that modulation of this prominent signaling pathway could provide a viable therapeutic approach for protection or recovery of neuronal circuits in PD and/or HD.

## Data Availability Statement

The raw data supporting the conclusions of this article will be made available by the authors, without undue reservation.

## Ethics Statement

All animal procedures were conducted according to the Animals Scientific Procedures Act UK (1986) and in compliance with the ethical standards at University College London (UCL).

## Author Contributions

PS conceived the overall project, guided the project, and provided the funding. AG contributed to the design and analyses of the electrophysiology experiments. SG performed the cell biology and behavioral experiments. SS performed the electrophysiology experiments and analyses of dendritic arborization. TD analyzed the electrophysiology data and helped in the preparation of figures. All authors participated in the design of experiments, interpretation of data, and writing of the manuscript.

## Conflict of Interest

The authors declare that the research was conducted in the absence of any commercial or financial relationships that could be construed as a potential conflict of interest.
