# Supplementary material for: Striatal Synapse Degeneration and Dysfunction Are Reversed by Reactivation of Wnt Signaling
Source: Front Synaptic Neurosci. 2021 Jun 3;13:670467. doi: 10.3389/fnsyn.2021.670467 (PMC8209303; doi:10.3389/fnsyn.2021.670467)
Supplement: Supplementary file 1 [file Data_Sheet_1.pdf]

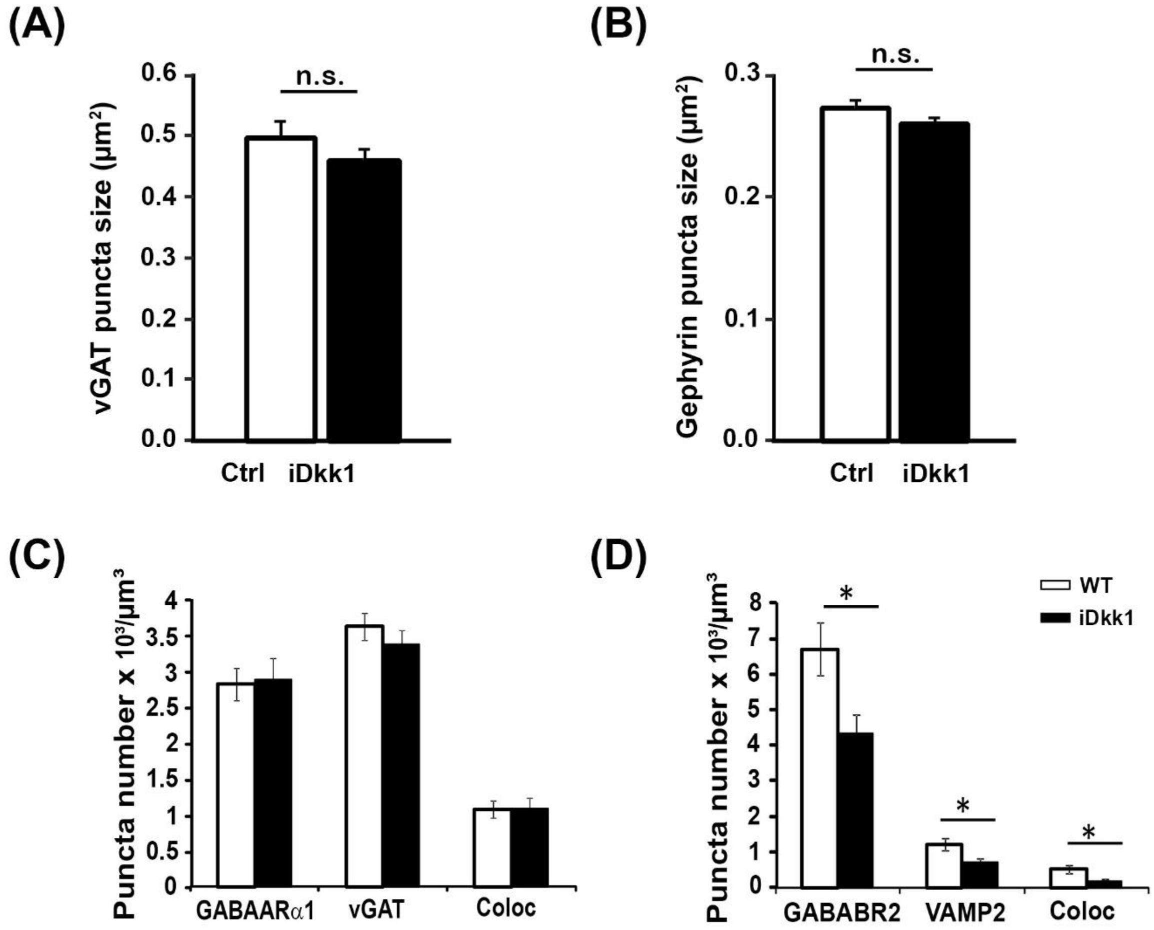

Figure Supplementary 1. Expression of Dkk1 does not affect the size of vGAT and Gephyrin puncta or the number of GABAARα1 but reduces the number of GABABR2 in the adult striatum. Confocal images of the striatum of mice exposed to Dkk1 expression for two weeks were used to evaluate volume of A) vGAT puncta and B) Gephyrin puncta and number of C) GABAARα1 and vGAT puncta as well as their colocalization D) GABABR2 and VAMP2 puncta as well as their colocalization. There was no significant difference due to genotype for vGAT and gephyrin puncta size and for number of GABAARα1 puncta ($p>0.05$) Mann-Whitney. However, GABABR2, VAMP2 puncta number and their colocalization was reduced in iDkk1 mice ($p<0.05$) Mann-Whitney. N=5 animals

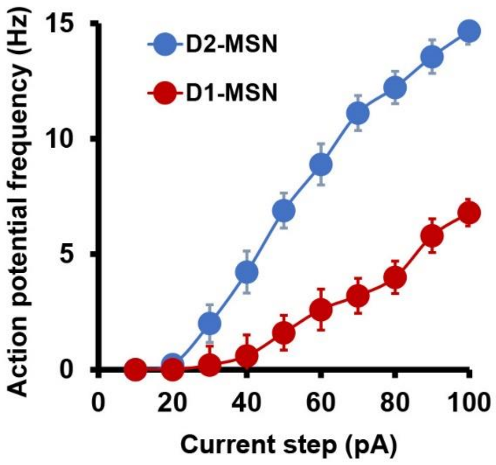

Figure Supplementary 2: Action potential frequency of D1R and D2R cells. The graph shows the different AP frequency of the two MSNs elicited by different current step intensities. These neurons were identified by the expression of GFP in the D2R transgenic mice and by the analysis of their morphology post recording. The AP frequency elicited by a given current step intensity are clearly distinct between the two cell populations and characteristic of these two types of neurons. N=9-10 cells from 5 animals
